# Induction of Autoimmune Myocarditis in Diversity Outbred Mice

**DOI:** 10.3390/biology15030288

**Published:** 2026-02-06

**Authors:** Meghna Sur, Kiruthiga Mone, Shraddha Singh, Mahima T. Rasquinha, Jean-Jack M. Riethoven, Indranil Mukhopadhyay, Raymond A. Sobel, Jay Reddy

**Affiliations:** 1 School of Veterinary Medicine and Biomedical Sciences, University of Nebraska-Lincoln, Lincoln, NE 68583, USA; mesur@coh.org (M.S.); kmone2@huskers.unl.edu (K.M.); ssingh22@huskers.unl.edu (S.S.); mahima.rasquinha@mssm.edu (M.T.R.); 2 Department of Immuno-Oncology, Beckmann Research Institute, City of Hope, Duarte, CA 91010, USA; 3 Department of Immunology and Immunotherapy, Icahn School of Medicine at Mount Sinai, New York, NY 10029, USA; 4 Nebraska Center for Biotechnology, University of Nebraska-Lincoln, Lincoln, NE 68588, USA; jeanjack@unl.edu; 5 Department of Statistics, University of Nebraska-Lincoln, Lincoln, NE 68583, USA; imukhopadhyay2@unl.edu; 6 Department of Pathology, Stanford University, Stanford, CA 94305, USA; raysobel@stanford.edu

**Keywords:** porcine cardiac myosin, myocarditis, mouse models, diversity outbred, immune cells, dilated cardiomyopathy

## Abstract

Background. Inbred mouse models of autoimmune myocarditis are routinely used to investigate the immune mechanisms underlying dilated cardiomyopathy. However, their translational relevance is limited because observations made in a single inbred strain may not reflect those of outbred human populations. This limitation can be overcome by using Diversity Outbred (DO) mice, whose genetic variability is comparable to that of humans. Methods. To investigate the utility of DO mice, we characterized their immune cell distributions and induced myocarditis by immunization with porcine cardiac myosin (PCM) emulsified in complete Freund’s adjuvant. Antigen-specific T cell and antibody responses were evaluated using lymphocytes and serum samples, respectively, and hearts were examined histologically for inflammatory changes. Results. First, we noted no significant variations in the majority of immune cell populations, which include T cells and B cells. However, NK cells, double positive for CD49b and NK1.1, were lacking in both sexes. While we noted sex differences in the expression of major histocompatibility complex class II molecules in antigen-presenting cells, expression of costimulatory molecules was similar in both sexes. Second, upon immunization, we demonstrated that the PCM was immunogenic, and the PCM-reactive T cell responses were generated in both males and females, as measured by a proliferation assay. Third, cytokine analysis revealed marginal detection of Th1 (IFN-γ) and Th17 (IL-17 and IL-22) cytokines, mainly with three doses of immunization. Fourth, determination of PCM-reactive antibody responses revealed significant amounts of IgG1 and IgG2b isotypes. Finally, histological analysis revealed varying degrees of myocarditis in individual mice of both sexes. Conclusions. Our data suggest that mild autoimmune myocarditis can be induced in DO mice. However, to capture the heterogeneity in disease susceptibility, large sample cohorts are required.

## Simple Summary

Researchers often use inbred mouse strains to study autoimmune diseases, including autoimmune myocarditis. These mice are genetically very similar to one another, which means that their responses may not reflect what happens in genetically diverse human populations. In this study, we examined whether Diversity Outbred (DO) mice, whose genetic composition more closely resembles that of humans, could be utilized for studying inflammatory processes in the heart. Our results showed that DO mice are capable of developing heart inflammation and indicate that the variations noted in our study may have translational significance.

## 1. Introduction

Myocarditis is the inflammation of the heart muscle that can lead to dilated cardiomyopathy (DCM). Various infectious and non-infectious triggers are implicated in the causation of myocarditis [1,2,3]. Recently, the occurrence of myocarditis has gained significant attention as immunocompetent individuals receiving vaccines, in particular for severe acute respiratory syndrome coronavirus 2, and cancer patients receiving checkpoint inhibitors have developed myocarditis as a side effect [4,5]. Thus, the determination of their underlying mechanisms is critical to understanding the pathogenesis of myocarditis and developing therapies.

The mouse models of experimental autoimmune myocarditis (EAM) are generally used since their histological features resemble those of DCM in humans, as evidenced by immune cell infiltrations, necrosis of cardiomyocytes, and fibrosis, leading to cardiac remodeling [6,7,8]. These models involve the use of inbred mouse strains. Essentially, the inbred mice are genetically identical, lacking human genetic diversity, and different inbred mouse strains exhibit different susceptibilities to myocarditis. For example, mice with a major histocompatibility complex (MHC) H-2^a^ background are highly susceptible to EAM, whereas those with an H-2^b^ background are resistant to the development of EAM [9]. Likewise, the EAM models are typically regarded as T cell-mediated disease, and autoantibodies are not critical for the progression of myocarditis [10,11]. In contrast, in humans, up to ~30% of those affected with myocarditis and DCM could carry autoantibodies [12,13,14,15,16]. As such, a single inbred strain is unlikely to capture the variation in human genetic predispositions and immune responses in the development of myocarditis. Thus, we asked whether the heterogeneity in immune response and disease susceptibility observed in Diversity Outbred (DO) mice recapitulates the variability seen in outbred human populations.

In that direction, DO mice have been recently created; they are derived from using eight inbred mouse strains, and their genetic diversity is comparable to that of outbred populations [17,18,19,20]. These include three classical laboratory strains (A/J, C57BL/6J, and 129S1/SvlmJ) that have been used extensively in biological research, including knockout models [21,22,23,24]; two mouse models for common human diseases, namely NOD/ShiLtJ for type I diabetes and NZO/HILtJ for obesity [25,26]; and three wild-derived mouse strains (CAST/EiJ, PWK/PhJ, and WSB/EiJ) [24,27,28,29]. We induced myocarditis using porcine cardiac myosin (PCM) and demonstrate the induction of both T cell and antibody responses specific to PCM.

## 2. Materials and Methods

### 2.1. Mice

Six-to-eight-week-old male and female DO mice were procured from the Jackson Laboratory (Bar Harbor, ME, USA). Mice were maintained according to the institutional guidelines of the University of Nebraska-Lincoln, Lincoln, NE, USA, and approved for animal studies by the university’s Institutional Animal Care and Use Committee (protocol #2294). Mice were housed up to five per cage in filter-top cages assembled with closed air circulation. Cages containing the chow diet and waterers were changed biweekly until the end of the experiment. The animals had ad libitum access to food and water during the entire study period. Euthanasia was performed using carbon dioxide as recommended by the Panel on Euthanasia of the American Veterinary Medical Association.

### 2.2. Immunophenotyping by Flow Cytometry

Spleens were harvested from DO mice, and single-cell suspensions were examined for the expression of immune cell-surface markers. Prior to staining, cells were treated with TruStain FcX™ (Biolegend, San Diego, CA, USA) and incubated for 15 min on ice. Then, the cells were stained for the expression of indicated markers (clone identifiers are indicated in parentheses): CD19 (clone 6D5; cat. no. 115424), CD80 (clone 16-10A1; cat. no. 104713), CD86 (clone GL-1; cat. no. 105017), F4/80 (clone BM8; cat. no. 123107), CD3 (clone 145-2C11; cat. no. 100325), CD11c (clone N418; cat. no. 117309), IA^b^ (clone KH74; cat. no. 115307), IA^k^ (clone 10-3.6; cat. no. 109905), IE^k^ (clone 14-4-4S; cat. no. 110205), CD8b (clone YTS156.7.7; cat. no. 126609), CD4 (clone GK1.5; cat. no. 100406), TCRγδ (clone GL3; cat. no. 118115), CD49b (clone DX5; cat. no. 108913), and NK1.1 (clone PK136; cat. no. 108725) (all from BioLegend). After incubation in the dark on ice for 15 min and washing twice, cells were acquired by flow cytometry (FACSCalibur™, BD Biosciences, Franklin Lakes, NJ, USA), and the percentages of different cell subsets were analyzed using FlowJo software v10.9 (Tree Star, Ashland, OR, USA).

### 2.3. Protein and Peptide Synthesis

The PCM (Sigma-Aldrich, St. Louis, MO, USA; cat. no. M0531) and keyhole limpet hemocyanin (KLH) (Sigma-Aldrich; cat. no. H7017) protein were commercially procured. Ovalbumin (Ova) 323–339 (VNTFVHESLADVQA) was synthesized on 9-fluorenylmethyloxycarbonyl chemistry (Genscript, Piscataway, NJ, USA) and purified by high-performance liquid chromatography (HPLC), and more than 90% purity was confirmed by HPLC and mass spectroscopy. Proteins and Ova 323–339 were dissolved in 1× phosphate-buffered saline (PBS), aliquoted, and stored at −20 °C.

### 2.4. Immunization Procedures

Immunizations involved PCM emulsified in complete Freund’s adjuvant (CFA) containing *Mycobacterium tuberculosis* H37RA extract (Difco Laboratories, Detroit, MI, USA) to obtain a final concentration of 5 mg/mL. The emulsions were administered subcutaneously (100 µg/mouse) in the left and right shoulders, the right and left sides of the hip regions, and the inguinal and sternum region in split doses on days 0 and 7. In some experiments, animals received the third dose of immunization on day 14. Additionally, animals received pertussis toxin (PT) (List Biological Laboratories, Campbell, CA, USA) intraperitoneally (500 ng/mouse) on days 0 and 2 after the first immunization. Of note, PT is generally used as an additional adjuvant that enhances disease induction by promoting innate immune activation, facilitating autoreactive T cell responses, and promoting infiltration of immune cells into target organs [30,31]. At terminations (day 21 or day 28), hearts were collected for histology, whereas spleen and draining lymph nodes were used to prepare lymphocytes for in vitro experimentation.

### 2.5. Proliferation Assay

Lymphocytes were stimulated with PCM or Ova 323–339 (0–100 µg/mL) at a concentration of 5 × 10^6^ cells/mL for two days in complete medium containing RPMI-1640 (HyClone, Logan, UT, USA; cat. no. SH30027.01) supplemented with 10% fetal calf serum (HyClone; cat. no. SH30070.03), 200 mM L-glutamine (Gibco, Waltham, MA, USA; cat. no. 25030081), 1 mM sodium pyruvate (Cytiva, Marlborough, MA, USA; cat. no. SH30239.01), 1× each of non-essential amino acids (HyClone; cat. no. SH30238.01), MEM vitamin mixture (Gibco; cat. no. 11120-052), 50 µM 2-mercaptoethanol (Sigma-Aldrich; cat. no. SHBPO744) and 100 U/mL penicillin–streptomycin (Lonza, Basel, Switzerland; cat. no. BW09-757F). Proliferative responses were measured as counts per minute (CPM) after pulsing with tritiated [^3^H] thymidine (1 µCi per well; Moravek Inc., Brea, CA, USA) for 16 h using a Wallac liquid scintillation counter (Perkin Elmer, Waltham, MA, USA).

### 2.6. Histopathology

For fixation, hearts were immersed in 10% phosphate-buffered formalin. Tissues were trimmed, processed overnight, and embedded in paraffin before being sliced into 5 µm-thick cross-sections and stained with hematoxylin and eosin (H&E). The sections were examined by a board-certified pathologist, blinded to treatment. Heart sections were evaluated for inflammatory foci, mineralization, and fibrosis [6,32,33]. We used the following grading scale: marked, severe or widespread inflammation; +, moderate inflammation; mild, perivascular inflammatory cells; mild (?), inflammatory cells within the vessel walls; and −, no abnormalities.

### 2.7. Determination of PCM-Reactive Antibodies

Serum samples obtained on day 0 and at termination (day 21) were analyzed for total Ig and antibody isotypes (IgM, IgG1, IgG2a, IgG2b, IgG2c, IgG3) by indirect enzyme-linked immunosorbent assay (ELISA). Polystyrene 96-well microtiter plates were coated with PCM or an irrelevant control (KLH) at a concentration of 0.5 μg/mL in 1×  PBS and incubated at 4 °C overnight. Plates were washed with 1×  PBS containing 0.05% Tween-20 (Polysorbate 20) (Fisher Scientific, Waltham, MA, USA; cat. no. BP337500) and blocked with 200 μL of assay buffer (1×  PBS containing 2% bovine serum albumin and 5% normal goat serum) for 1.5 h at room temperature. Serum samples diluted 1:100 were then added in duplicates and incubated at 37 °C for 1 h. After washing, horseradish peroxide (HRP)-labeled goat anti-mouse total Ig, IgM, IgG1, IgG2a, IgG2b, and IgG3 (cat. no. 5300-05), as well as IgG2c (cat. no. 1079-05), diluted 1:500 in assay buffer, were added as secondary antibodies (Southern Biotech, Birmingham, AL, USA). The plates were incubated at room temperature for 2 h, followed by washing and the addition of 100 μL of 1×  tetramethylbenzidine substrate solution (Rockland Immunochemicals, Pottstown, PA, USA; cat. no. TMBE-RED-1000). Reactions were stopped using 1 M phosphoric acid, and the optical density (OD) values were measured at 450 nm using an automated ELISA reader (BioTek Instruments, Winooski, VT, USA).

### 2.8. Cytokine Bead Array Analysis

Lymphocytes were obtained from DO mice immunized with PCM. Cells were stimulated with PCM or Ova 323–339 (10–100 µg/mL), and after 48 h, the culture supernatants were collected. Cytokines were analyzed using the LEGENDplex Murine Th cytokine Panel (12-plex; BioLegend, cat. no. 741043). The panel consisted of interferon (IFN)-γ, interleukin (IL)-2, IL-4, IL-5, IL-13, IL-9, IL-17A, IL-17F, IL-22, IL-6, tumor necrosis factor (TNF)-α, and IL-10. The lyophilized mouse cytokine standard mix provided in the kit was serially diluted to obtain the standard curve. The capture beads/cytokine antibody conjugates were added, followed by the addition of detection antibodies and streptavidin–phycoerythrin reagents to the diluted standards and test samples. Beads were acquired by flow cytometry, and cytokine concentrations were determined using the LEGENDplex™ data analysis software suite, version 2023-02-15 (BioLegend).

### 2.9. Statistical Analysis

All graphs were prepared using GraphPad Prism software v8.1.0 (GraphPad Software, Inc., La Jolla, CA, USA). For phenotypic expression analysis, the Shapiro–Wilk test was used to check for normality [34], with the majority of data, for each proliferative response and for DO males and females, resulting in *p* < 0.05, which indicates non-normally distributed data. Therefore, analyses were performed using non-parametric statistical tests. For single-factor analysis of variance, Kruskal–Wallis was utilized [35], while for two-factor analysis, Sheirer–Ray–Hare was applied [36]. For significant omnibus tests, Dunn’s test with Benjamini–Hochberg multiple-test correction was used [37,38]. To determine differences between DO males and females, the Wilcoxon rank-sum test was used [39]. Data on proliferative response were analyzed using a mixed model with replication as random effects and concentration levels as fixed effects. An F-test was used to detect the significance of the concentration effect. When significance in the concentration effects was noted, pairwise comparisons were performed using a paired *t*-test. Datasets on cytokine bead array analysis and antibody titers were measured using the Wilcoxon signed-rank test and paired *t*-test, respectively, for paired samples. Where standard error mean (SEM) values are shown, error bars indicate the scatteredness of the mean data. *p*-value ≤ 0.05 is considered statistically significant.

## 3. Results

### 3.1. The Distribution of the Majority of Immune Cell Subsets Was Comparable Between Male and Female DO Mice

We analyzed the splenocyte subsets corresponding to T cells and non-T cells, which included the antigen-presenting cells (APCs; dendritic cells [DCs], CD11c; macrophages, F4/80; B cells, CD19), with respect to the MHC class II and costimulatory molecules (Supplementary Figures S1–S7 and Supplementary Tables S1 and S2).

First, by gating the CD3^+^ subset, we analyzed T cell populations, namely CD4, CD8, γδ-T, and natural killer (NK)-T (CD49b^+^NK1.1^+^), and their proportions did not differ significantly between DO males and females (Supplementary Figure S2 and Supplementary Table S1). A similar analysis in the CD3^−^ subset for B cells (CD19^+^), macrophages (F4/80^+^), and DCs (CD11c^+^) also revealed no significant differences between sexes (Supplementary Figure S3 and Supplementary Table S1). However, NK cells expressing both CD49b^+^NK1.1^+^ were absent in both sexes of DO mice. We next analyzed the expression of MHC class II and costimulatory molecules in the APCs. We considered four MHC class II alleles, IA^b^, IA^k^, IA^g7^, and IE^k^, using either IA^b^ (clone, KH74), IE^k^ (clone, 14-4-4S), or IA^k^ (clone, 10-3.6) antibodies, of which IA^k^ cross-reacted with IA^g7^. We found that female DO mice expressed only IA^k^/IA^g7^ on APCs, but APCs from male DO mice expressed IA^k^/IA^g7^, IE^k^, and IA^b^ (Supplementary Figures S4–S6 and Supplementary Table S2). These analyses led us to note that all three APCs (B cells, DCs, and macrophages) from DO males consistently expressed IA^b^, IA^k^/IA^g7^, and IE^k^ molecules in variable frequencies. In contrast, the female DO mice expressed mainly IA^k^/IA^g7^, whereas the expression of IA^b^ and IE^k^, if any, was negligible (Supplementary Table S2). Although overall expression of IA^b^ (*p* < 0.01) and IE^k^ (*p* < 0.01) was higher in the DO males than that of females, no significant variations were noted with any of the above molecules when comparisons were made within the individual APC subsets (B cells, macrophages, and DCs) (Supplementary Figure S3 and Supplementary Table S1). Likewise, by evaluating the expression of costimulatory molecules, namely CD80 and CD86, we did not observe any significant variations (Supplementary Figure S7 and Supplementary Table S2).

### 3.2. DO Mice Immunized with PCM Generate Antigen-Specific T Cell Responses

We asked whether the DO mice, which are genetically unique and whose diversity captures the variation in outbred human populations, can develop myocarditis. We used PCM, which has been previously shown to induce myocarditis in inbred mouse strains [40,41,42,43]. Furthermore, we could not use the mouse cardiac myosin peptides because their use is only relevant to a specific inbred mouse strain. For example, cardiac myosin heavy chain (myhc)-α 334–352 induces myocarditis in A/J mice [6], and myhc-α 614–643 induces myocarditis in BALB/c mice [7]. Since DO mice can express a range of MHC molecules representing eight different strains, it becomes difficult to predict which peptide might be appropriate [17,18,19,20]. Furthermore, generally, myocarditis is induced with two immunizations, and rarely, three immunizations were also used, as in the case of cardiac troponin I (cTnI) [44]. Thus, we used both two-time and three-time immunization procedures to investigate the ability of DO mice to develop myocarditis.

**Figure 1:**
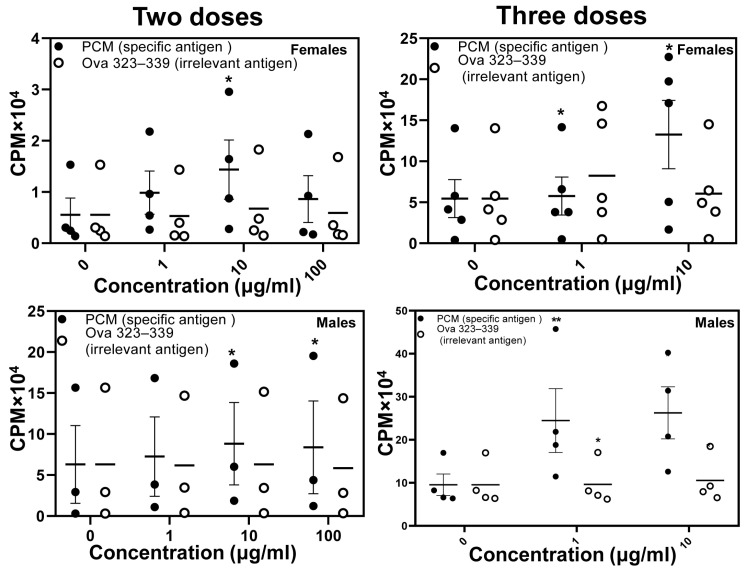
Lymphocytes from PCM-immunized DO mice respond to the protein antigen specifically. Lymphocytes were harvested from animals immunized twice (**left panel**) or thrice (**right panel)** separately at termination. Cells were stimulated with or without PCM and Ova 323–339 (control). After two days, whole cells were pulsed with [^3^H] thymidine for 16 h, the incorporation of which was measured as CPM. Error bars indicate the SEM values obtained from three to five mice per group. After the overall significance of concentration effects was detected through mixed model analysis, pair-wise comparison was done using a *t*-test. * *p* ≤ 0.05 and ** *p* ≤ 0.01.

By immunizing groups of males and females, we initially determined the T cell responses using PCM and Ova 323–339 as specific and irrelevant antigens, respectively, in a recall proliferation assay. The analysis revealed that lymphocytes harvested from both females and males responded to PCM but not OVA in a dose-dependent manner (Figure 1, left panels). The proliferative responses in females were marginally higher (~3-fold) compared to males (~2-fold). Similar trends were observed when lymphocytes from animals immunized three times were analyzed (Figure 1, right panels). While these data support the idea that PCM was immunogenic and induced T cell responses, we asked whether the immunized animals also produced PCM-reactive antibodies, as the myocarditis-inducing ability of cardiac antigens is attributed to T cell responses in inbred mouse strains.

### 3.3. Immunization with PCM Results in the Production of Mainly IgG Responses Specific to PCM

**Figure 2:**
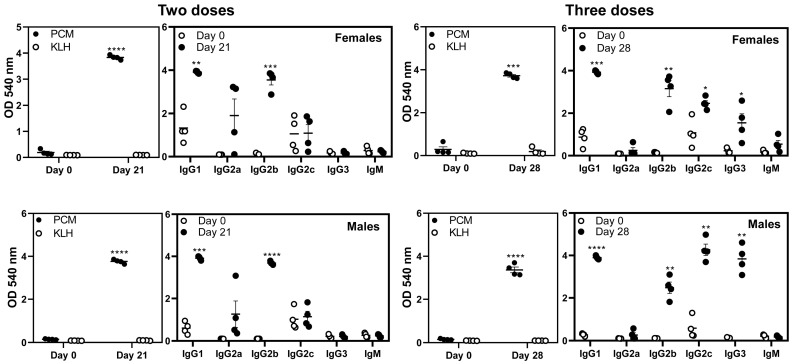
Antibody responses induced by DO mice immunized with PCM primarily included the IgG isotypes. Serum was collected from the indicated groups on day 0, day 21, or day 28 separately. The samples were diluted (1:100) and added in duplicates to high-binding plates previously coated with PCM or KLH (control). After adding HRP-conjugated goat anti-mouse total Ig (**left panel**), IgM, IgG1, IgG2a, IgG2c, and IgG3 (**right panel**) as detection antibodies, reactions were stopped. Plates were read at 450 nm to obtain the OD values. The right panels in each setting (two doses and three doses) indicate reactivity to PCM. Error bars indicate the SEM values obtained from four mice of each group. Paired *t*-test was used to determine the significance between groups, i.e., day 0 vs. day 21 and day 0 vs. day 28, and the data were analyzed separately. * *p* ≤ 0.05, ** *p* ≤ 0.01, *** *p* ≤ 0.001, and **** *p* ≤ 0.0001.

To analyze antibodies, we established an ELISA in which KLH was used as an irrelevant antigen and PCM as a specific antigen, permitting us to capture antigen-specific responses. The data indicated that animals of both sexes immunized twice developed PCM-reactive antibodies but not for KLH (female, *p* = 0.0000298; male, *p* = 0.00000385), as indicated by the measurement of total IgG (Figure 2, left panels). Since total Ig reactivity was lacking for KLH, we did not include this antigen for determining antibody isotypes. By testing for different isotypes, we noted that the antibodies of IgG1 and IgG2b were significantly elevated in both sexes (female, *p* = 0.00588; male, *p* = 0.0003177). Although similar trends were noted with IgG2a, the differences were not significant, whereas IgG3 and IgM levels were not detected. We then contrasted these antibody profiles with the animals immunized thrice with PCM, leading us to note significant elevations in the total IgG (Figure 2, right panels). As expected, the levels of IgG1 and IgG2b also increased, similar to the animals immunized twice with PCM (Figure 2, left and right panels). Strikingly, however, while the trends of IgG2a were comparable between animals immunized twice or thrice, IgG2c (female, *p* = 0.04; male, *p* = 0.0014) and IgG3 (female, *p* = 0.054; male, *p* = 0.001429) levels were significantly elevated only in the animals immunized thrice with PCM, whereas IgM levels were unchanged (Figure 2, right panels). These data suggest that repeat immunizations potentiate the induction of a broad range of IgG isotypes that may also be influenced by the genetic makeup of each DO mouse.

### 3.4. Cytokine Analysis Revealed No Significant Differences in Response to PCM Immunization in DO Mice

**Figure 3:**
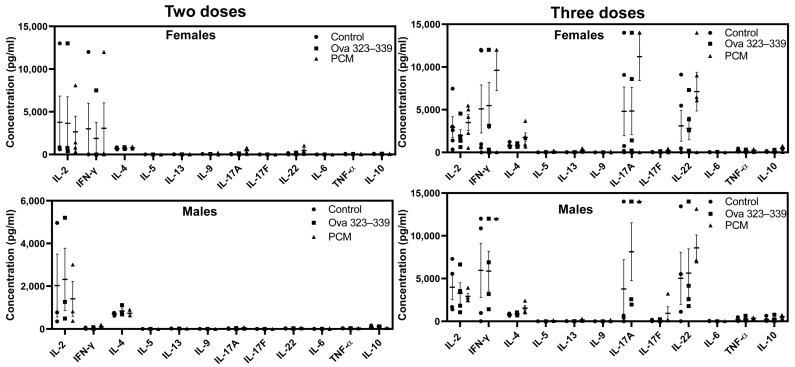
Lymphocytes obtained from DO mice immunized with PCM thrice tended to produce Th1 and Th17 cytokines. Lymphocytes were obtained from animals immunized with PCM twice or thrice separately at termination. Cells were stimulated with or without PCM or Ova 323–339 (50 µg/mL), and supernatants harvested on day 2 were analyzed for indicated cytokines using LEGENDplex Murine Th cytokine Panel (12-plex; BioLegend) as described in the Methods section. Error bars indicate the SEM values obtained from three to five mice. We used the Wilcoxon signed-rank test for paired samples to determine significance between groups.

To determine cytokine responses, we used multiplex bead array analysis on the culture supernatants obtained from lymphocytes stimulated with or without PCM or Ova 323–339. The cytokine analysis of the DO mice of either sex immunized twice with PCM revealed no major trends in the production of cytokines of various Th subsets (Figure 3, left panels). The lack of cytokine secretion appears not to be due to a lack of T cell proliferation since lymphocytes from PCM-immunized mice responded antigen specifically (Figure 1, left panels). In addition, splenocytes stimulated with anti-CD3 as a polyclonal activator revealed that T cells from both male and female DO mice responded to anti-CD3 (*p* < 0.05), and if any, the responses were relatively higher in male compared to female DO mice (Supplementary Figure S8). However, upon analyzing the cytokines in animals immunized thrice, we observed that the culture supernatants from both sexes contained Th1 (IFN-γ) and Th17 (IL-17A and IL-22) cytokines but with no significant differences (Figure 3, right panels).

### 3.5. DO Mice Immunized with PCM Develop Mild Myocarditis

**Figure 4:**
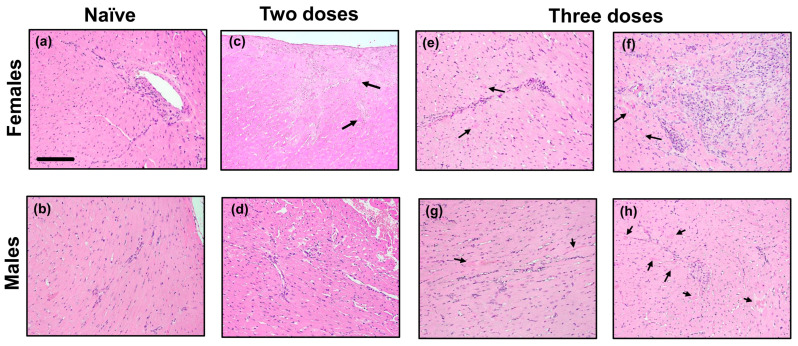
DO mice develop mild myocarditis in response to PCM immunization. DO mice immunized with PCM twice or thrice were euthanized on day 21 or day 28, respectively, and hearts were collected for histological assessment by H&E staining. Naïve DO mice, panels (**a**,**b**); DO mice immunized with PCM twice, panels (**c**,**d**); and DO mice immunized with PCM thrice, panels (**e**–**h**). Arrows indicate individual necrotic myocytes. Original magnification, ×20 (bar = 100 µm, applies to all panels). Representative images are shown from three naïve and four to five immunized mice for each male and female groups.

We examined hearts for myocarditis severity by H&E staining. Generally, myocarditis-affected mice do not exhibit clinical illness, and the DO mice of both sexes, immunized twice or thrice, remained clinically normal and did not lose body weight. Of note, heart sections from one naive female mouse had inflammatory cells within the vessel walls, which was graded as mild (?), whereas the other had mild inflammation (Figure 4a). Similarly, heart sections from two naïve male mice revealed inflammatory cells within the vessel wall, graded as mild (?) (Figure 4b). Animals immunized twice were euthanized on day 21, whereas those immunized thrice were euthanized on day 28, and the hearts were examined for inflammatory changes. Heart sections from one of each of the female and male mice immunized twice showed mild infiltrates along with individual myocyte necrosis (Figure 4c,d). In a similar analysis of heart sections from animals immunized thrice, three out of five (60%) females showed mild to marked inflammation (Figure 4e,f). Similarly, two out of four males (50%) had mild inflammatory infiltrates (Figure 4g,h). Nonetheless, other changes expected in myocarditis-affected animals of the inbred mouse strains, such as myocardial necrosis, fibrosis, and mineralization, were absent.

## 4. Discussion

DO mice have been used to study the influence of genetic factors on disease susceptibility [45], physiology [46,47], autoimmunity [48,49], metabolism [50,51], vaccine response [52,53], behavior [54,55], and toxicity studies [56,57]. Due to the paucity of immunological information available on DO mice, we first sought to verify the distribution of immune cell populations. Although most immune subsets were comparable between sexes, NK cells double positive for CD49b and NK1.1 were absent in both DO males and females. Of note, strain differences have been noted in the expression of NK cell markers in that C57BL/6 and SJL mice express NK1.1, NKp46, and CD49b, whereas BALB/c, NOD, and 129 mice do not express NK1.1 [58,59,60,61]. Additional characterization of NK cell markers may be necessary to determine the utility of DO mice for NK cell research. We noted sex-associated differences in MHC class II expression in DO mice. Females primarily expressed IA^k^/IA^g7^ MHC class II molecules, whereas males additionally expressed IE^k^ and IA^b^. This likely reflects sex-dependent expression of MHC molecules. For example, estrogens promote the upregulation of MHC class II molecules as opposed to downregulation with testosterone [62]. However, expression patterns of costimulatory molecules were similar in both sexes.

After determining the distribution of immune cells, we sought to determine the utility of DO mice for the induction of autoimmune diseases. We chose the EAM model as the relevant tools were readily available to analyze PCM-reactive responses. Of note, various autoimmune myocarditis models have been developed to investigate the immune mechanisms of cardiac autoimmunity [63,64,65]. These involve immunization with cardiac proteins (murine myhc-α or PCM and cTnI) [40,41,42,43,63,64,66] or their immunogenic peptides emulsified in CFA using inbred mouse strains. For example, murine myhc-α 334–352/CFA emulsion can induce severe myocarditis in A/J mice bearing the H-2^a^ MHC haplotype, and the chronically affected animals can develop DCM [6]. However, the same peptide can induce myhc-α 334–352-specific T cell responses in C57BL/6J mice bearing the MHC haplotype H-2^b^, but they are resistant to the development of severe myocarditis [63]. On the other hand, murine myhc-α 614–643 induces myocarditis in susceptible BALB/c mice expressing the H-2^d^ haplotype, which also develop DCM [7]. Likewise, other peptides, such as cTnI 105–122 [44], sarcoplasmic/endoplasmic reticulum Ca^2+^ (SERCA2a) 971–990, β1-adrenergic receptor 211–230, branched-chain α-ketoacid dehydrogenase kinase 111–130, and adenine nucleotide translocator 1 21–40, have also been shown to induce myocarditis in A/J mice [67,68,69,70]. Furthermore, while the majority of the above EAM models in susceptible mice are associated with inflammatory infiltrates, myocardial necrosis, fibrosis, and mineralization, we noted that SERCA2a 971–990 immunization induces mainly atrial myocarditis in A/J mice [67]. These observations suggest that inducing myocarditis in a specific mouse strain is similar to analyzing the disease process in a single human. Thus, due to the lack of genetic diversity in inbred mouse strains, establishing myocarditis models in outbred strains may be translationally relevant. To that end, we used PCM that was previously shown to induce myocarditis similar to mouse cardiac myosin [40,41,42,43]. We did not use the well-characterized epitopes listed above because DO mice can express a range of MHC molecules derived from eight different strains [17,18,19,20], making it difficult to predict which peptide might be appropriate.

By evaluating the T cell response to PCM, we noted a marginal increase in females as compared with males, especially with two doses of immunization, but no such difference was observed with three doses. PCM was used previously to induce myocarditis in mouse strains expressing MHC class II IA^k^ and IE^k^ (A/J and C3H), including H2^d^ (BALB/c) [40,41,42,43]. The finding that the female DO mice expressed IA^k^ and IE^k^ could suggest that the immunodominant epitopes of PCM might have been preferentially presented by these molecules. Conversely, male DO mice expressed MHC class II/IA^b^ molecule in addition to the above, and the inbred C57BL/6J mice expressing IA^b^ molecule are regarded as a myocarditis-resistant strain [63,64,65]. Thus, we speculate that the broader MHC class II expression observed in male DO mice might have diversified antigen presentation in the context of wider MHC molecules, potentially reducing the relative magnitude of any single antigen-specific T cell response. This could also explain comparable myhc-α 334–352-reactive T cell responses noted in female and male DO mice after three immunizations. That is, the frequencies of antigen-primed T cells generated in response to two doses could be low in male DO mice, and the third immunization might have led to their expansion. However, this variation in antigen-specific T cell responses appears not to be due to a defect in the ability of T cells to respond to stimuli because the T cell responses to anti-CD3 stimulation were noted in both sexes.

We next investigated antibody responses to PCM, leading us to note a difference between two- and three-dose immunizations. While PCM-reactive IgG1 and IgG2b antibodies were significantly elevated after two doses, IgG2c and IgG3 were found to be increased only after three doses. Induction of IgG1, IgG2a, and IgG2b in response to PCM, correlating with the development of myocarditis, has been previously reported as evaluated in a two-time immunization protocol [40]. Furthermore, the induction of a broad range of IgG isotypes is worth noting, as DO mice are genetically diverse, carrying genomes from A/J, C57BL/6J, and NOD mice, among others [54]. IgG2c is found in two of the eight founders of DO mice, namely C57BL/6J and NOD, whereas IgG2a, IgG2b, IgG1, and IgG3 are commonly reported in various other mouse strains [71]. Although the use of DO mice offers an advantage to capture breadth of different isotypes, as we had previously noted in response to vaccines [72], the differential upregulation (IgG1 and IgG2b with two doses vs. IgG2c and IgG3 with three doses of PCM) suggests that their detection could be influenced by T cell cytokines [73]. We noted the detection of Th1 and Th17 cytokines in response to three doses of PCM immunization in both sexes. However, no definitive conclusions can be made due to variations observed between individual immunized animals within each group. Nonetheless, various T cell cytokines have been shown to promote isotype switching of various classes: IL-4 for IgG1 [32]; IFN-γ for IgG2a, IgG2c, and IgG3 [74]; IL-21 for IgG1 and IgG2b [75]; IL-17A for IgG2a and IgG3 [76]; and TGF-β for IgG2b and IgG3 [77]. In our cytokine analysis, we noted the detection of IFN-γ, IL-17A, and, to a lesser extent, IL-4, and we did not test for other cytokines listed above; their determination would be helpful to correlate with the production of different isotypes.

Finally, by histological evaluation of hearts, we noted that DO mice immunized twice had only mild myocarditis. Because that one naive female mouse also had mild inflammation, it is difficult to relate whether two immunizations could promote disease induction. However, ~50% of animals receiving three doses developed myocarditis, and if any, females tended to develop marked inflammation. The lack of severe myocarditis may be due to insufficient production of inflammatory Th1 (IFN-γ) and Th17 family cytokines (IL-17A and IL-22) that are shown to be critical for the induction of myocarditis [78]. Additionally, other cytokines such as IL-6 and TNF-α, including chemokines (macrophage inflammatory protein (MIP)-1α, MIP-2, and C-X-C motif chemokine ligand 1) and granulocyte–macrophage colony-stimulating factor, have also been implicated [79,80,81]. In our panel, IL-6 and TNF-α were not detected, whereas others were not tested. Furthermore, the role of IFN-γ and IL-4 in the induction of myocarditis seems unclear, as their blockade led to disease induction and protection phenotypes in A/J mice, respectively, leading to a suggestion that myocarditis might be associated with Th2 cytokines [32]. Therefore, clarity is needed to demonstrate the role of T cell cytokines in the induction of myocarditis in the DO model, which can be addressed by comprehensively analyzing various cytokines and chemokines using advanced technologies such as Luminex, Olink Proximity Extension Assay, single-molecule array, and CyTOF [82,83,84,85,86]. Nonetheless, the lack of severe myocarditis noted is unlikely to be due to the production of immune regulatory cytokines, such as IL-10, which was not significantly increased in our studies.

## 5. Conclusions

In this brief report, we described the cellular distributions of immune cells in DO mice and determined their immunogenicity to PCM in the induction of myocarditis. The major findings include sex-dependent expression of MHC class II molecules and lack of expression of CD49b^+^NK1.1^+^ NK cells; a tendency for females to respond to PCM; variations in the detection of antibody isotypes and cytokines; and disease occurrence depending on the frequencies of immunizations. Because the genetic diversity of DO mice is comparable to that of humans, the observations made in our studies may be translationally relevant, as each DO mouse is unique due to its fixed genetic makeup [87]. Furthermore, not all individuals affected with myocarditis-associated triggers, such as viruses, drugs, or toxins, develop myocarditis, indicating broad host-dependent variability [88,89,90]. However, several limitations of this study preclude definitive conclusions. First, sample sizes were small, and DO mice are generally used in quantitative genetics and the identification of quantitative trait loci to investigate the genetic basis for disease susceptibility [24]. Thus, it is possible to capture more diverse disease phenotypes using large cohorts of mice. Alternatively, the collaborative cross (CC) mice derived from the same eight inbred mouse founders as those of DO mice may be more suitable for studying the disease phenotypes [91,92,93]. Because the fixed genetic diversity is derived from close to 200 recombinations per CC line, which are also inbred, their use offers high mapping power in which diverse disease phenotypes could be captured reproducibly. Second, although the use of irrelevant antigens was helpful to capture antigen (PCM)-specific T cell reactivity and antibodies, a more comprehensive analysis of cytokines may be helpful to correlate T cell and antibody responses. While adoptive transfer experiments have historically been helpful for determining the roles of antigen-specific T cell and antibody responses in inbred mouse strains, their execution in DO mice is challenging due to potential alloreactivity. Third, the myocarditis induction protocols did not involve adjuvants alone groups, namely CFA, PT, and a combination of both. This is critical because heart muscle sections from naïve DO mice showed individual myocyte necrosis, which may confound interpretation. Finally, the duration of this study is short, where the immunized animals were euthanized to ascertain inflammatory changes as a readout. While myocarditic animals appear clinically normal, the use of serum cardiac injury markers, such as cTnI and creatine kinase-MB, would be helpful for characterizing the disease process. Likewise, even with large cohorts of mice, DO mice are expected to show heterogeneity in myocarditis development. However, a critical question that needs to be addressed is how many of those could develop DCM because estimates indicate that ~20% of the individuals affected with myocarditis can develop DCM [94,95]. Thus, the use of DO mice could serve as an ideal platform for mapping genetic determinants of susceptibility and resistance to DCM development. In that setting, the use of non-invasive tools such as echocardiography or magnetic resonance imaging would be helpful to ascertain structural and functional abnormalities of myocardial function [96,97]. Overall, the data from this brief report in DO mice provide insights into genetic susceptibility to myocarditis that may have relevance to humans. However, larger cohorts, appropriate controls, and additional diagnostic tools will be needed to make more definitive conclusions.

## Data Availability

The original data presented in this study are openly available in the article/Supplementary Material and at the URL (https://doi.org/10.71964/125). Further inquiries can be directed to the corresponding author.

## Supplementary Materials

The following supporting information can be downloaded at: https://www.mdpi.com/article/10.3390/biology15030288/s1. Figure S1: Gating strategy used for immunophenotyping. Splenocytes were stained with antibodies for the indicated markers. After acquiring the cells by flow cytometry, the percentage of cells positive for each marker was analyzed using the FlowJo software v10.9; Figure S2: Determination of the distribution of T cell subsets in DO mice. Splenocytes from male and female DO mice were stained with antibodies for the indicated markers following Fc receptor blocking. After acquiring the cells by flow cytometry, cells positive for CD3 were gated, in which the percentages of cells positive for CD4, CD8, γδ, NK1.1, and CD49 were analyzed using FlowJo software v10.9 (*n* = 3); Figure S3: Determination of the distribution of non-T cell subsets in DO mice. Splenocytes from male and female DO mice were stained with antibodies for the indicated markers following Fc receptor blocking. After acquiring the cells by flow cytometry, cells negative for CD3 were gated, in which the percentages of cells positive for CD19, F4/80, CD11c, NK1.1, and CD49b were analyzed using FlowJo software v10.9 (*n* = 3); Figure S4: Determination of the expression of MHC class II molecule IA^b^ in the antigen-presenting cells in DO mice. Splenocytes from male and female DO mice were stained with antibodies for the indicated markers following Fc receptor blocking. After acquiring the cells by flow cytometry, the percentages of cells positive for IA^b^ were analyzed in relation to CD19 (B cells), F4/80 (macrophages), and CD11c (dendritic cells) markers using FlowJo software v10.9 (*n* = 3); Figure S5: Determination of the expression of MHC class II molecule IA^k^ in the antigen-presenting cells in DO mice. Splenocytes from male and female DO mice were stained with antibodies for the indicated markers following Fc receptor blocking. After acquiring the cells by flow cytometry, the percentages of cells positive for IA^k^ were analyzed in relation to CD19 (B cells), F4/80 (macrophages), and CD11c (dendritic cells) markers using FlowJo software v10.9 (*n* = 3); Figure S6: Determination of the expression of MHC class II molecule IE^k^ in the antigen-presenting cells in DO mice. Splenocytes from male and female DO mice were stained with antibodies for the indicated markers following Fc receptor blocking. After acquiring the cells by flow cytometry, the percentages of cells positive for IE^k^ were analyzed in relation to CD19 (B cells), F4/80 (macrophages), and CD11c (dendritic cells) markers using FlowJo software v10.9 (*n* = 3); Figure S7: Determination of the expression of costimulatory molecules (CD80 and CD86) in the antigen-presenting cells in DO mice. Splenocytes from male and female DO mice were stained with antibodies for the indicated markers following Fc receptor blocking. After acquiring the cells by flow cytometry, cells negative for CD3 were gated, in which the percentages of cells positive for CD80 and CD86 were analyzed in relation to CD19 (B cells), F4/80 (macrophages), and CD11c (dendritic cells) markers using FlowJo software v10.9 (*n* = 3); Figure S8: Proliferative response of splenocytes to anti-CD3. Splenocytes from male and female DO mice were stimulated with anti-CD3 for two days. After pulsing for 16 h with tritiated [^3^H] thymidine, proliferative responses were measured. Mean ± SEM values representing three mice are shown. Statistical analysis was performed as described in the methods section (Kruskal–Wallis followed by Dunn with Benjamini–Hochberg). * *p* < 0.05; Table S1: Cellular distribution of splenocytes in DO mice; Table S2: Expression of MHC class II and costimulatory molecules in antigen-presenting cells from DO mice.

## Abbreviations

The following abbreviations are used in this manuscript:

APCs	Antigen-presenting cells
CC	Collaborative cross
CFA	Complete Freund’s adjuvant
CPM	Counts per minute
cTNI	Cardiac troponin I
DC	Dendritic cell
DCM	Dilated cardiomyopathy
DO	Diversity outbred
EAM	Experimental autoimmune myocarditis
ELISA	Enzyme-linked immunosorbent assay
HPLC	High-performance liquid chromatography
IFN	Interferon
IL	Interleukin
KLH	Keyhole limpet hemocyanin
myhc	Myosin heavy chain
NK	Natural killer
OD	Optical density
Ova	Ovalbumin
PCM	Porcine cardiac myosin
