# Supplementary material for: Induction of Autoimmune Myocarditis in Diversity Outbred Mice
Source: Biology (Basel). 2026 Feb 6;15(3):288. doi: 10.3390/biology15030288 (PMC12896637; doi:10.3390/biology15030288)
Supplement: Supplementary file 1 [file biology-15-00288-s001.zip › biology-4070294-supplementary.pdf]

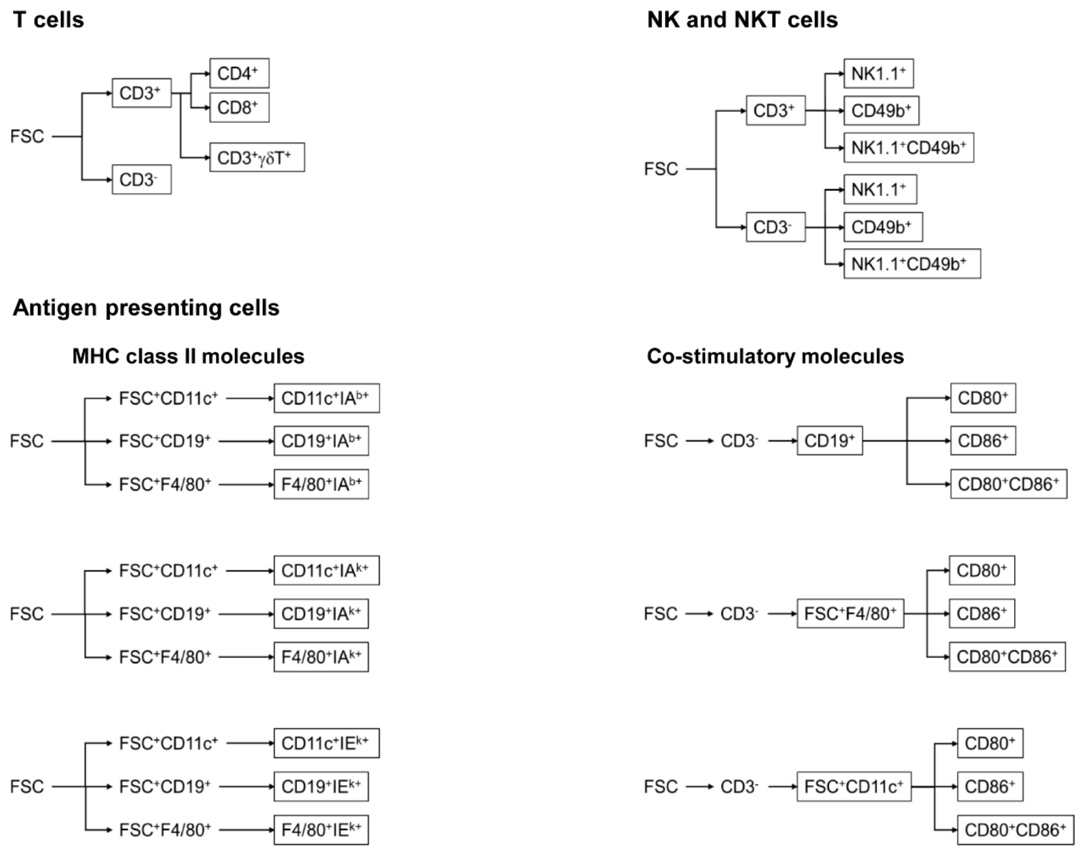

Figure S1: Gating strategy used for immunophenotyping. Splenocytes were stained with antibodies for the indicated markers. After acquiring the cells by flow cytometry, the percentage of cells positive for each marker was analyzed using the FlowJo software v10.9.

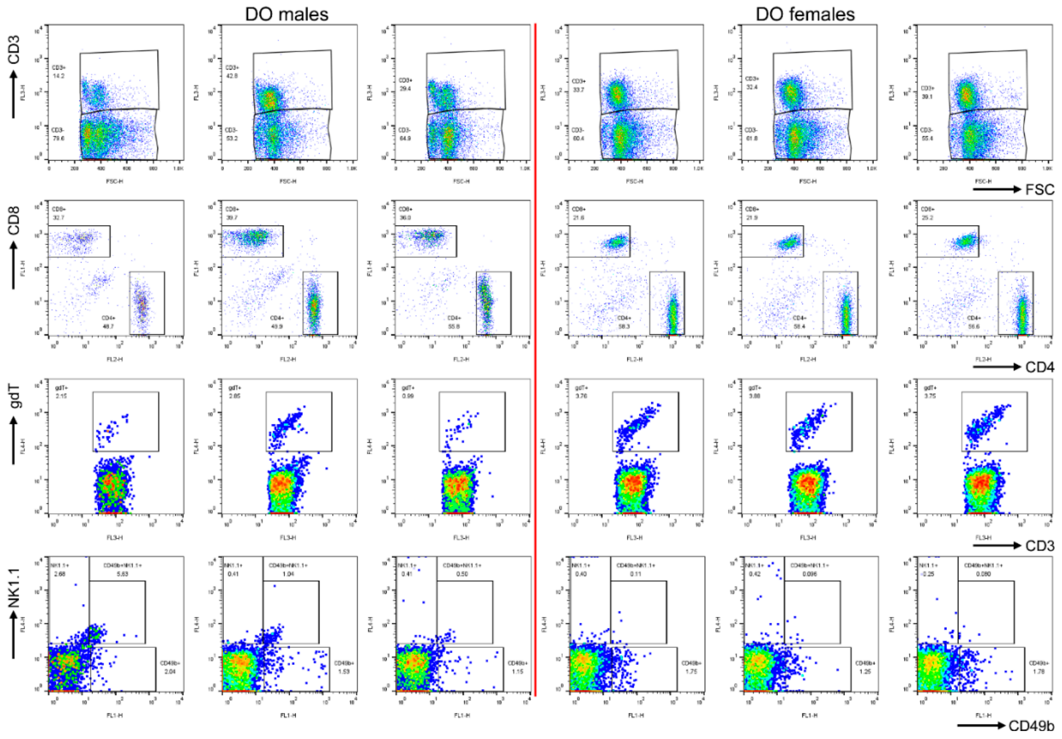

Figure S2: Determination of the distribution of T cell subsets in DO mice. Splenocytes from male and female DO mice were stained with antibodies for the indicated markers following Fc receptor blocking. After acquiring the cells by flow cytometry, cells positive for CD3 were gated, in which the percentages of cells positive for CD4, CD8, γδ, NK1.1, and CD49 were analyzed using FlowJo software v10.9 (*n*=3).

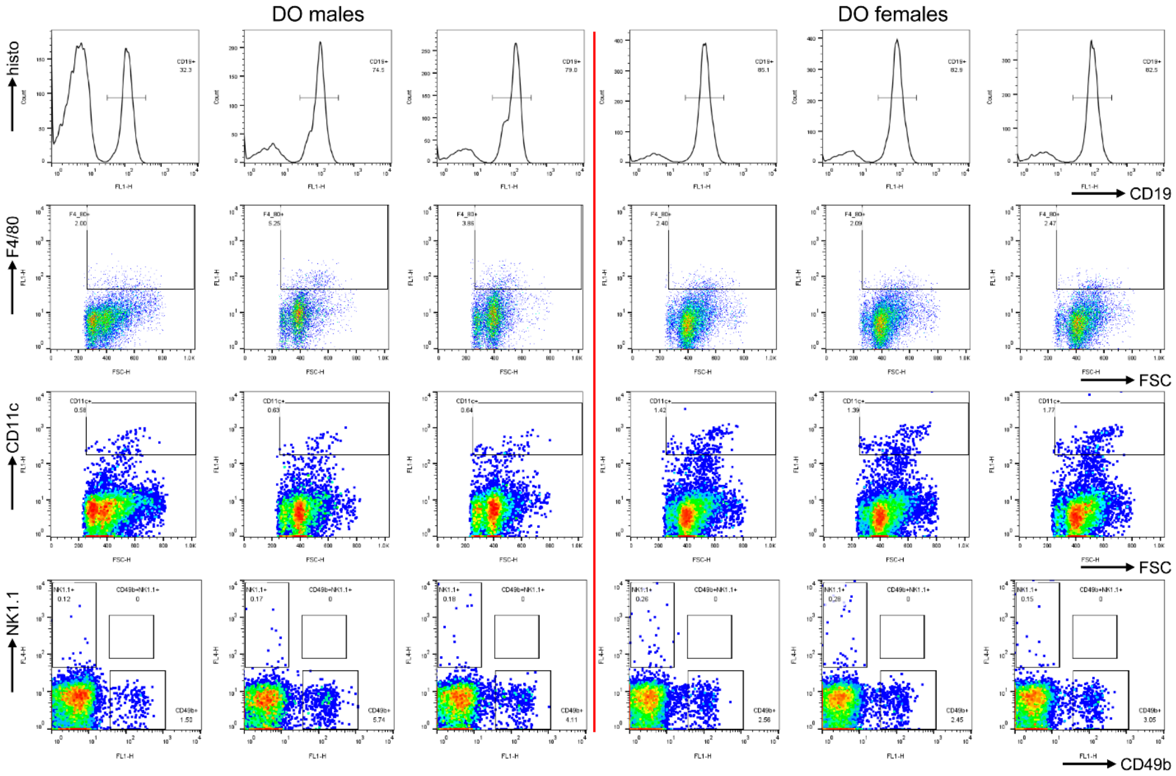

Figure S3: Determination of the distribution of non-T cell subsets in DO mice. Splenocytes from male and female DO mice were stained with antibodies for the indicated markers following Fc receptor blocking. After acquiring the cells by flow cytometry, cells negative for CD3 were gated, in which the percentages of cells positive for CD19, F4/80, CD11c, NK1.1, and CD49b were analyzed using FlowJo software v10.9 (*n*=3).

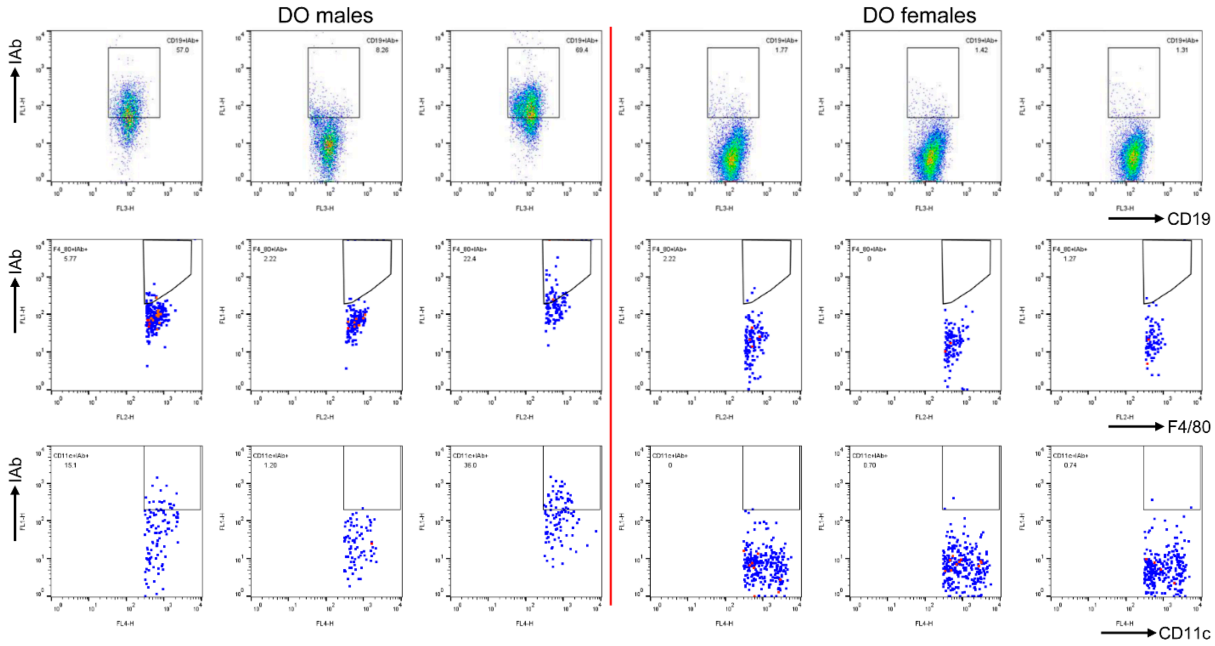

Figure S4: Determination of the expression of MHC class II molecule, IA^{b}, in the antigen-presenting cells in DO mice. Splenocytes from male and female DO mice were stained with antibodies for the indicated markers following Fc receptor blocking. After acquiring the cells by flow cytometry, in which the percentages of cells positive for IA^{b} were analyzed in relation to CD19 (B cells), F4/80 (Macrophages), and CD11c (Dendritic cells) markers using FlowJo software v10.9 (*n*=3).

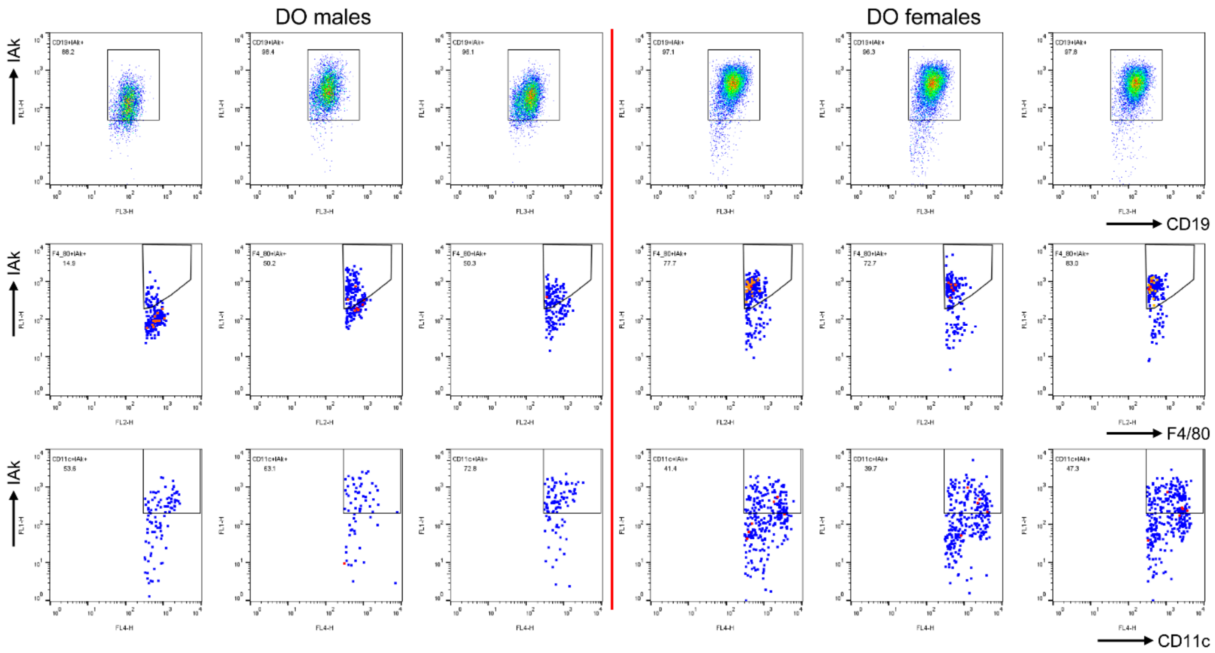

Figure S5: Determination of the expression of MHC class II molecule, IA^k, in the antigen-presenting cells in DO mice. Splenocytes from male and female DO mice were stained with antibodies for the indicated markers following Fc receptor blocking. After acquiring the cells by flow cytometry, in which the percentages of cells positive for IA^k were analyzed in relation to CD19 (B cells), F4/80 (Macrophages), and CD11c (Dendritic cells) markers using FlowJo software v10.9 (*n*=3).

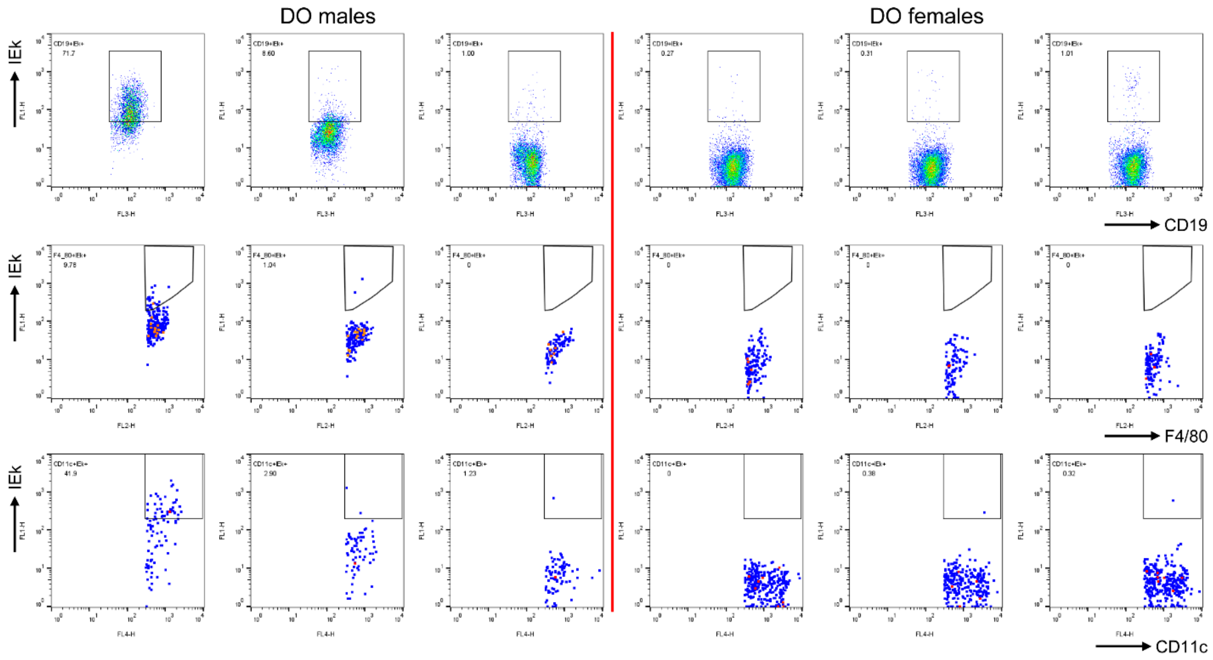

Figure S6: Determination of the expression of MHC class II molecule, IE^k, in the antigen-presenting cells in DO mice. Splenocytes from male and female DO mice were stained with antibodies for the indicated markers following Fc receptor blocking. After acquiring the cells by flow cytometry, in which the percentages of cells positive for IE^k were analyzed in relation to CD19 (B cells), F4/80 (Macrophages), and CD11c (Dendritic cells) markers using FlowJo software v10.9 (*n*=3).

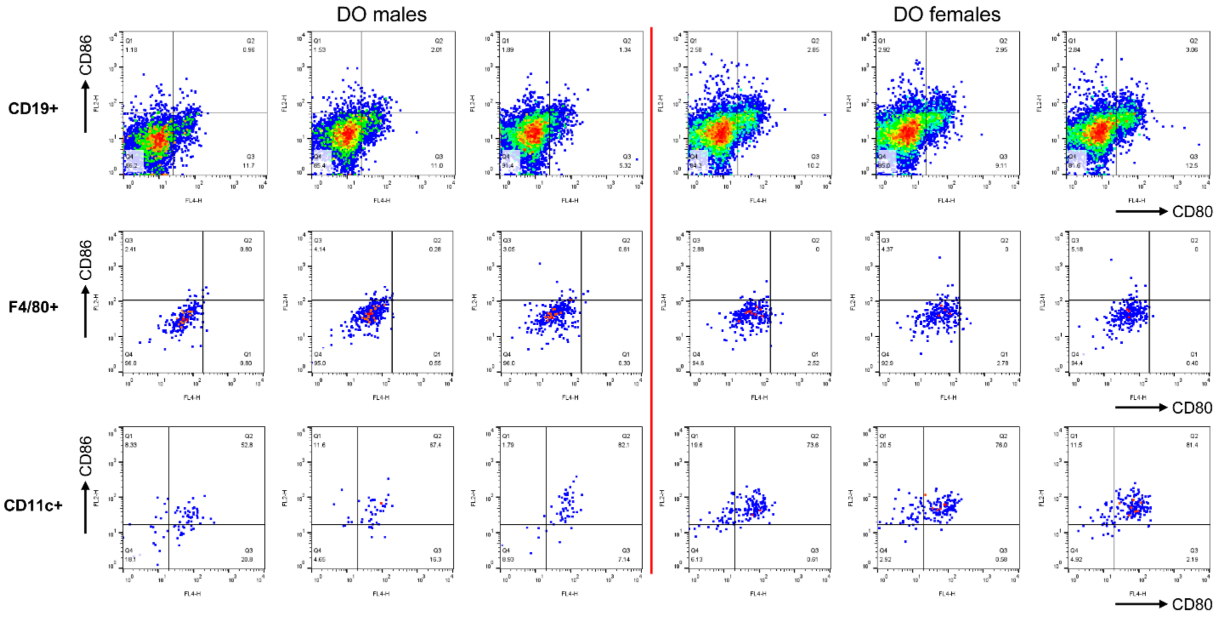

Figure S7: Determination of the expression of costimulatory molecules (CD80 and CD86) in the antigen-presenting cells in DO mice. Splenocytes from male and female DO mice were stained with antibodies for the indicated markers following Fc receptor blocking. After acquiring the cells by flow cytometry, cells negative for CD3 were gated, in which the percentages of cells positive for CD80 and CD86 were analyzed in relation to CD19 (B cells), F4/80 (Macrophages), and CD11c (Dendritic cells) markers using FlowJo software v10.9 (*n*=3).

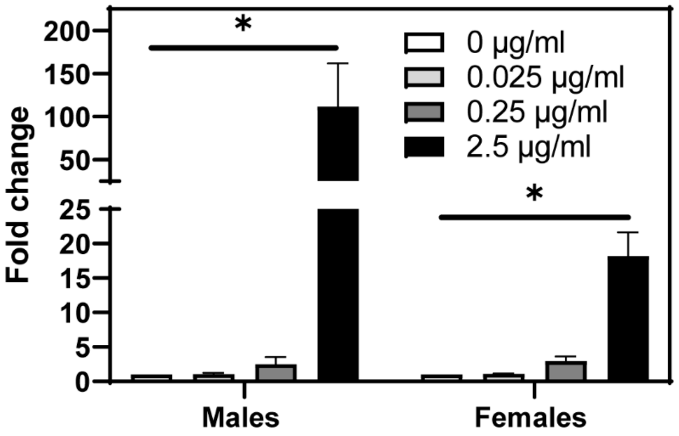

Figure S8: Proliferative response of splenocytes to anti-CD3. Splenocytes from male and female DO mice were stimulated with anti-CD3 for two days. After pulsing for 16 hours with tritiated [^{3}H] thymidine, proliferative responses were measured. Mean ± SEM values representing three mice are shown. Statistical analysis was performed as described in the methods section (Kruskal-Wallis followed by Dunn with Benjamini-Hochberg). $^*p < 0.05$.

Table S1: Cellular distribution of splenocytes in DO mice

Subset	Males	Females
**T cells**		
$CD3^+$ cells	28.8 ± 6.7	37.0 ± 1.9
CD4	51.5 ± 1.8	56.0 ± 0.4
CD8	36.1 ± 1.7	23.0 ± 0.9
γδT	2.0 ± 0.4	3.8 ± 0.1
NK-T		
CD49b	1.6 ± 0.2	1.7 ± 0.1
NK1.1	1.2 ± 0.6	0.3 ± 0.0
$CD49b^+$ $NK1.1^+$	2.4 ± 1.3	0.1 ± 0.0
**Non-T cells**		
$CD3^-$ cells	65.9 ± 6.2	59.2 ± 1.9
CD19	61.9 ± 12.1	83.5 ± 0.7
F4/80	3.7 ± 0.8	2.3 ± 0.1
CD11c	0.6 ± 0.0	1.5 ± 0.1
NK		
CD49b	3.8 ± 1.0	2.7 ± 0.1
NK1.1	0.1 ± 0.0	0.3 ± 0.03
$CD49b^+$ $NK1.1^+$	0.0 ± 0.0	0.0 ± 0.0

Table S2: Expression of MHC class II and co-stimulatory molecules in antigen-presenting cells from DO mice

Sex	Subset	IA^b	IA^{k*}	IE^k	CD80	CD86	$CD80^+CD86^+$
Males	B cells	44.9 ± 15.2	94.2 ± 2.5	27.1 ± 18.3	9.3 ± 1.6	1.5 ± 0.2	1.4 ± 0.3
	Macrophages	10.1 ± 5.1	38.5 ± 9.6	3.6 ± 2.5	0.6 ± 0.1	3.2 ± 0.4	0.6 ± 0.1
	Dendritic cells	17.4 ± 8.3	63.2 ± 4.5	15.3 ± 10.8	14.7 ± 3.3	7.2 ± 2.4	67.4 ± 6.9
Females	B cells	1.5 ± 0.1	97.1 ± 0.4	0.5 ± 0.2	10.6 ± 0.8	2.8 ± 0.1	3.0 ± 0.0
	Macrophages	1.2 ± 0.5	77.8 ± 2.4	0.0 ± 0.0	1.9 ± 0.6	4.1 ± 0.5	0.0 ± 0.0
	Dendritic cells	0.5 ± 0.2	42.8 ± 1.9	0.2 ± 0.1	1.1 ± 0.4	17.2 ± 2.3	77 ± 1.9

*α-IA^k antibody cross-reacts with IA^{g7}
